# Genome-Wide Identification and Expression Analysis of *BnPP2C* Gene Family in Response to Multiple Stresses in Ramie (*Boehmeria nivea* L.)

**DOI:** 10.3390/ijms242015282

**Published:** 2023-10-18

**Authors:** Yu Chen, Haohan Zhao, Yue Wang, Xiaojun Qiu, Gang Gao, Aiguo Zhu, Ping Chen, Xiaofei Wang, Kunmei Chen, Jia Chen, Peng Chen, Jikang Chen

**Affiliations:** 1 Institute of Bast Fiber Crops, Chinese Academy of Agricultural Sciences, Changsha 410221, China; cy17630961283@163.com (Y.C.); zhaohaohan@caas.cn (H.Z.); 15269595618@163.com (Y.W.); qiuxjstu@163.com (X.Q.); gaogang@caas.cn (G.G.); zhuaiguo@caas.cn (A.Z.); chenping02@caas.cn (P.C.); wangxiaofei@caas.cn (X.W.); chenkunmei@caas.cn (K.C.); chenjia01@caas.cn (J.C.); 2 College of Agriculture, Guangxi University, Nanning 530004, China; 3 National Breeding Center or Bast Fiber Crops, MARA, Changsha 410221, China

**Keywords:** protein phosphatase 2C (PP2C), ramie, abscisic acid (ABA), evolutionary analysis, expression patterns

## Abstract

The protein phosphatase 2C (*PP2C*), a key regulator of the ABA signaling pathway, plays important roles in plant growth and development, hormone signaling, and abiotic stress response. Although the *PP2C* gene family has been identified in many species, systematic analysis was still relatively lacking in ramie (*Boehmeria nivea* L.). In the present study, we identified 63 *BnPP2C* genes from the ramie genome, using bioinformatics analysis, and classified them into 12 subfamilies, and this classification was consistently supported by their gene structures and conserved motifs. In addition, we observed that the functional differentiation of the *BnPP2C* family of genes was restricted and that fragment replication played a major role in the amplification of the *BnPP2C* gene family. The promoter *cis*-regulatory elements of *BnPP2C* genes were mainly involved in light response regulation, phytohormone synthesis, transport and signaling, environmental stress response and plant growth and development regulation. We identified *BnPP2C* genes with tissue specificity, using ramie transcriptome data from different tissues, in rhizome leaves and bast fibers. The qRT-PCR results showed that the *BnPP2C1*, *BnPP2C26* and *BnPP2C27* genes had a strong response to drought, high salt and ABA, and there were a large number of stress-responsive elements in the promoter region of *BnPP2C1* and *BnPP2C26*. The results suggested that *BnPP2C1* and *BnPP2C26* could be used as the candidate genes for drought and salt tolerance in ramie. These results provide a reference for further studies on the function of the *PP2C* gene and advance the development of the mechanism of ramie stress response, with a view to providing candidate genes for the molecular breeding of ramie for drought and salt tolerance.

## 1. Introduction

The type 2C protein phosphatases (*PP2Cs*) belong to a branch of phosphoprotein metal phosphatases (PPM), which are Mg^2+^ or Mn^2+^ dependent protein molecules and are widely found in plants and animals, especially in greater abundance in plants [1]. The *PP2C* is highly conserved during evolution and has a unique protein structure with an active catalytic region at the C-terminal or N-terminal end, and a structurally diverse extension region at the other end [2]. The structural diversity of the extension region allows for the diversification of *PP2C* genes and confers different biological functions to *PP2C* family genes [2]. In higher plants, *PP2C* plays an important role in plant growth and development, hormone signaling and abiotic stress responses [3,4].

The *PP2C* families have been reported in many plant species, such as *Arabidopsis thaliana* (80) [5], wheat (*Triticum aestivum*) (257) [6], cucumber (*Cucumis sativus*) (56) [7], walnut (*Juglans regia*) (44) [8], etc. Phylogenetic analysis classified the 80 *AtPP2C* genes in *Arabidopsis* into 13 subfamilies (A-L), while the other seven were not involved in the grouping. *PP2C* is a direct target of ABA receptor proteins (PYR/PYL/RCAR) in the ABA signaling pathway, and the interaction between PYR/PYL/RCAR proteins and *PP2C* activates downstream targets [9]. All of the nine members belonging to subgroup A of *AtPP2Cs* contain ABA-responsive elements in their promoter regions, among which ABA-insensitive 1 (ABI1) and ABA-insensitive 2 (ABI2) act by negatively regulating ABA signaling, and can also directly inhibit PP2C activity in vitro [10]. The B-branch members of *PP2C* genes mainly function in the mitogen-activated protein kinase (MAPK) signaling pathway, acting as negative regulators of plant hormone synthesis and defenses, and usually inactivating MPK4 and MPK6 for signal transduction regulation [11,12]. *PP2C* C-branch member and D-branch member genes regulate plant flowering development and salinity stress response, respectively [13,14]. It has been reported that many *PP2C* members are involved in the regulation of abiotic stresses such as high salt and drought in plants. For example, in *Betula alba*, overexpression of *BpPP2C1* significantly enhanced the salt tolerance of plants [15]; *PP2C49* negatively regulates plant salt tolerance by inhibiting the activity of *Arabidopsis AtHKT1;1* [16]. *ZmPP84*, a member of the *PP2C* F-branch, negatively regulates plant drought tolerance by affecting maize stomatal closure and thereby inhibiting the *ZmMEK1*–*ZmSIMK1* signaling pathway [17].

Ramie (*Boehmeria nivea* L.), a perennial fiber crop of the genus *Boehmeria* in the family Urticaceae, has been widely used as material for animal feed, the textile industry and the phytoremediation of heavy metal contaminated soil [18,19]. Ramie is one of the most important economic crops in Asia, and has been cultivated for over 4000 years [20]. Its production is governed by various abiotic and biotic stress conditions, such as drought, salt, and nutrient stresses. Drought stress is one of the major threats to ramie production, and under severe drought stress, ramie yield will be lost by more than 20% [20,21]. Several studies have reported that drought triggered the biosynthesis of ABA and induced leaf abscission and yield reduction in ramie [22]. Therefore, it is important to know the molecular mechanisms of ramie’s response to stresses. However, as one of the key regulators of the ABA signaling pathway, the *BnPP2C* gene family in ramie has not been studied previously.

Based on the genomic and transcriptomic data of ramie in our laboratory, this study was conducted to identify and analyze the gene family of *BnPP2C* using bioinformatics methods, and to analyze their expression patterns under stressful environments, with the aim of providing a reference basis for subsequent studies on the functions of *BnPP2Cs*, advancing the development of stress resistance mechanisms in ramie, thereby contributing to the genetic improvement of ramie for a resistant cultivar.

## 2. Results

### 2.1. Identification and Basic Information of PP2C Family Members in Ramie

In this study, a total of 63 *PP2C* family members from the whole genome database of ramie were identified, and were named *BnPP2C1* to *BnPP2C63*, based on their distribution in chromosomes. Physicochemical property analysis showed that the protein sequences of the *BnPP2C* gene family members were highly variable, among which *BnPP2C10* had the shortest amino acid sequence of 164 aa, and *BnPP2C28* had the longest amino acid sequence of 1088 aa. The molecular weights of these *BnPP2C* proteins ranged from 17.95 kDa to 121.62 kDa, with PIs ranging from 4.66 to 9.63, including 14 basic proteins and 49 acidic proteins (Supplementary Table S1).

The predicted results of subcellular localization showed that 27 of the 63 *BnPP2C* members were localized in chloroplasts, 19 in the cytosol, and 12 in the nucleus; *BnPP2C28* and *BnPP2C38* were found in the endoplasmic reticulum, and *BnPP2C56*, *BnPP2C55* and *BnPP2C53* were found in the cytoskeleton, vascular membrane and mitochondrion, respectively (Supplementary Table S1).

### 2.2. Phylogenetic Tree Analysis

**Figure 1:**
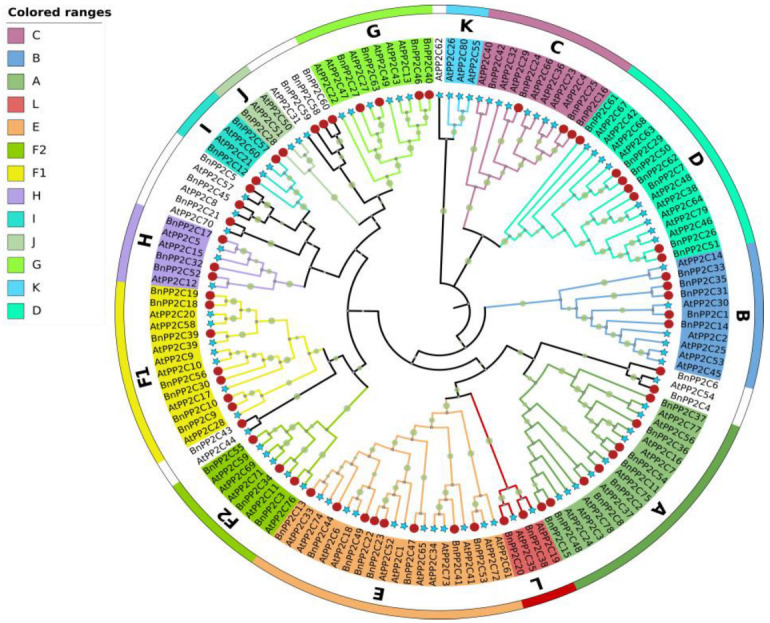
The phylogenetic tree of *BnPP2C* proteins and *AtPP2C* proteins. The *BnPP2Cs* are labeled with red circles and *AtPP2Cs* with blue stars. Different colors represent different subfamilies.

To investigate the phylogenetic relationship between ramie and *Arabidopsis PP2C* genes, a phylogenetic tree of 63 ramie *BnPP2C* and 80 *Arabidopsis AtPP2C* proteins were constructed using the neighbor joining method by MEGA11.0 software (Figure 1). The results showed that the 63 genes of the ramie *PP2C* family could be divided into 12 subfamilies, with subgroup A and subgroup E having the most *BnPP2C* members (8), followed by subgroup D and F1 (7), in the phylogenetic tree. Combining the data on the physicochemical properties of *BnPP2C*, it was found that the physicochemical properties of the proteins differed significantly among the subfamilies, while small differences were found within the same subfamily. Interestingly, none of the *BnPP2C* genes have been categorized into subgroup K, and no details have been reported on the function of *PP2C* subfamily K in other species.

### 2.3. BnPP2C Gene Structure Analysis and Conserved Motif Analysis

**Figure 2:**
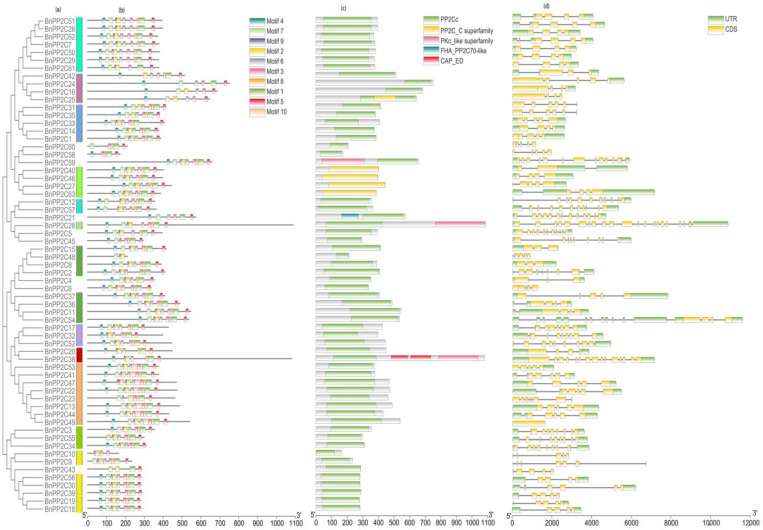
The phylogenetic tree, conserved motifs, conserved domain, and gene structure of the *PP2C* gene family in ramie. (**a**) The neighbor joining tree with 1000 bootstrap replicates of all BnPP2C proteins. (**b**) The composition of conserved motifs in *PP2C* proteins of ramie. (**c**) Conserved structure of BnPP2C identified by the NCBI Batch CD-search tool. (**d**) The gene structure of BnPP2C genes. The green blocks are CDS, yellow blocks are UTR.

The conserved motifs, conserved structural domains, coding sequences (CDS) and the location of the untranslated region (UTR) of *BnPP2C* were analyzed (Figure 2). We integrated and visualized the evolutionary tree and motif prediction results of the *BnPP2C* family using TBtools software and used the MEME online website to predict the conserved motifs of the *BnPP2C* family members. The results revealed some differences in the type and number of conserved motifs contained in each protein sequence. In the same sub-family species, the *BnPP2C* members had similar conserved motif structures. The results suggested that most *BnPP2C* genes were evolutionarily conserved, and individual members might be functionally divergent.

The structural domain analysis of *BnPP2C* family genes revealed that all 63 *BnPP2C* family gene members had *PP2C* structural domains, among which five genes contained *PP2C-C* superfamily structural domains. In addition, *BnPP2C59*, *BnPP2C28* and *BnPP2C38* had PKc-like superfamily structural domains, which might play important roles in the signal transduction cascade reaction.

Different gene structures affected gene expression and protein function, and exon–intron structural changes played an important role in gene functional differentiation [12,23]. Based on the phylogenetic analysis of *BnPP2C*, it was found that members of the same subgroup had similar exon and intron structures, and there were significant differences in intron and exon structures among members of different subgroups; for example, *BnPP2C49* had only one intron, but *BnPP2C28* had 20 introns.

### 2.4. Chromosome Distribution and Gene Duplication of BnPP2Cs

**Figure 3:**
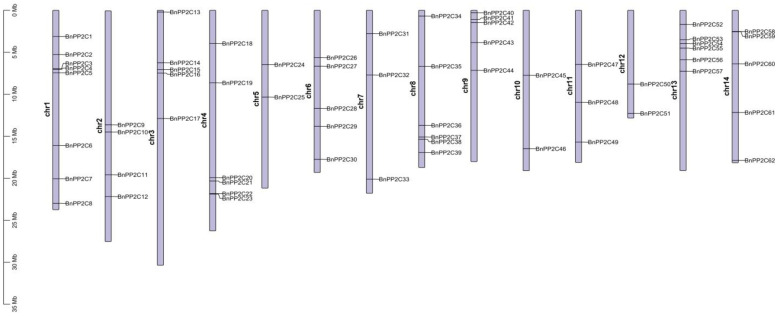
Chromosome distribution of the *PP2C* gene family in ramie. The left scale indicates the chromosome length (Mb).

After identification of the ramie *PP2C* gene family, 63 members of the *BnPP2C* family were mapped on the chromosomes by TBtools and renamed according to the chromosome number and the order of the position of each gene on the chromosome (*BnPP2C1-BnPP2C63*). The analysis showed that the 62 *BnPP2C* genes assembled were unevenly distributed on the 14 chromosomes of ramie (Figure 3), with most *BnPP2Cs* anchored in Chr1 (8). Only two *BnPP2Cs* in each were anchored in Chr5, Ch10 and Chr12. All of the *BnPP2C* genes located on Chr9 were distributed in the first half of the chromosome.

**Figure 4:**
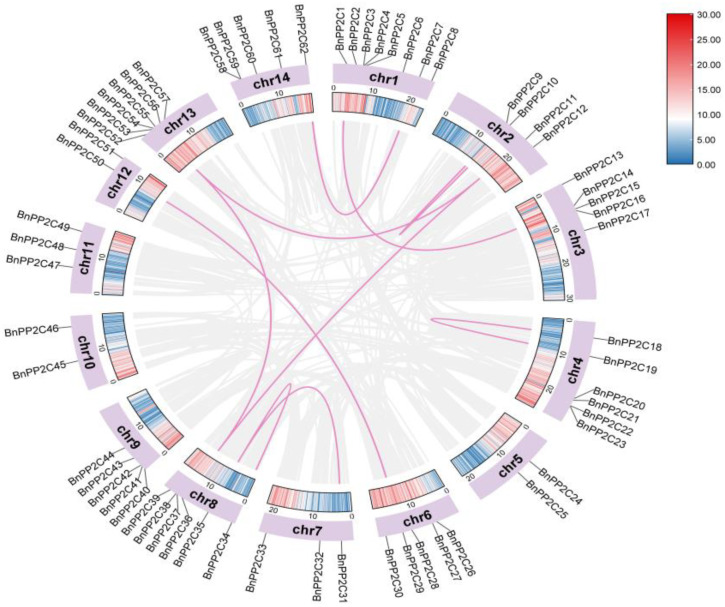
The collinearity analysis of *BnPP2Cs*. The gray lines indicate all co-linear gene pairs in ramie, and red lines indicate co-linear gene pairs in *BnPP2Cs*. The color on the chromosome represents the gene density, with red indicating the highest density and blue the lowest density.

**Figure 5:**
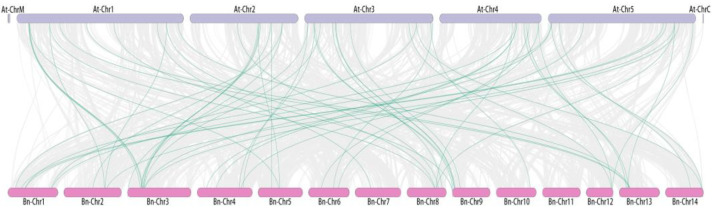
The collinearity analysis of *BnPP2Cs* and *AtPP2Cs*. The gray lines indicate all co-linear gene pairs between ramie and *Arabidopsis*, and the green lines indicate co-linearity of ramie *PP2Cs* with *Arabidopsis*.

To further understand the amplification mechanism of *BnPP2C*, the collinearity of *PP2C* in the ramie genome was investigated (Figure 4; Supplementary Table S2). The results revealed that there were 10 co-linear gene pairs among members of the ramie *PP2C* gene family, with three pairs of tandem duplicated genes distributed on Chr2, Chr4 and Chr8, respectively. The remaining seven pairs of segmentally duplicated genes were distributed unevenly on the remaining chromosomes, except Chr4, Chr5, Chr9, Chr10 and Chr11, where *BnPP2C36*, *BnPP2C11* and *BnPP2C54* were mutually segmentally duplicated gene pairs. In addition, we performed collinearity analysis on ramie and *Arabidopsis* and detected 59 homologous gene pairs, among which no homologous genes with *Arabidopsis* were present on ramie Chr11 and the most *PP2C* homologous genes (11) were present on ramie Chr3 (Figure 5; Supplementary Table S2).

The Ka/Ks value determines whether the gene encoding the protein is under selection pressure, which has an important role in the evolutionary analysis of gene families (Ka/Ks > one for positive selection, Ka/Ks = one for neutral selection, and Ka/Ks < one for purifying selection) [24,25] (Supplementary Table S3). The analysis showed that, except for two gene pairs, *BnPP2C7–BnPP2C62* and *BnPP2C34–BnPP2C35*, which were significantly differentiated and evolved over a long distance, the Ka/Ks values of all gene pairs were less than 1, indicating that the evolutionary process of these genes evolved mainly under purifying selection [26].

### 2.5. Cis-Acting Elements Analysis

**Figure 6:**
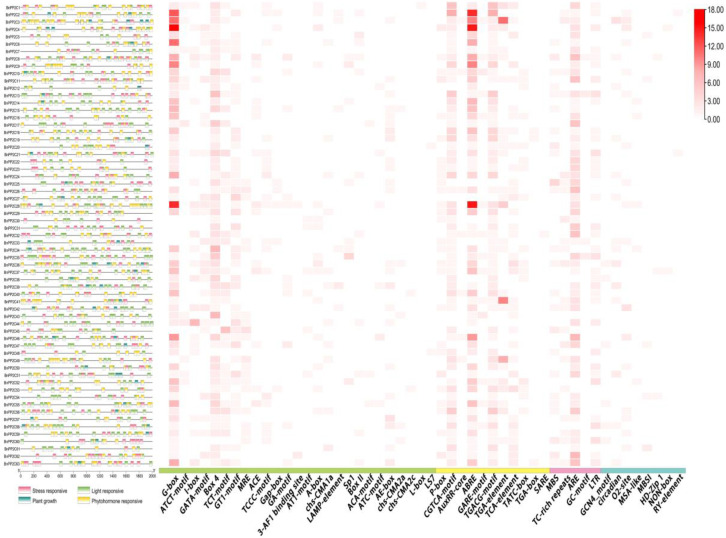
*Cis*-regulatory element analysis of the *BnPP2C* genes. Note: The different colors on the left represent the different elements (hormone response elements, light response elements, stress response elements and plant growth related elements) and the distribution of the 50 acting elements in the 63 *BnPP2C* genes is shown in the heat map on the right.

To further clarify the potential function of the *BnPP2C* gene family, *cis*-acting elements in the upstream 2 kb region were analyzed (Figure 6; Supplementary Table S4). We realized that abundant responsive regulatory elements were found in the promoter region of *BnPP2C*, and a total of 50 *cis* elements were detected, which were mainly classified into four categories. The first category included the most responsive light-responsive elements (26), such as G-box, Box4, etc. The second category included the hormone-responsive elements (11), such as P-box, ABRE, etc. The third category included responsive stress response elements (5), such as MBS, ARE, etc. The fourth category included plant growth response elements (8), such as O2-site, circadian, etc. 

Calculation of the number of elements revealed that the light-responsive element G-box and the hormone-responsive element ABRE had the largest number. The ARE element was the most distributed among the five stress elements, but the plant growth-responsive elements were present in very small numbers. Among them, G-box, Box4, ABRE and ARE elements were mainly distributed in *BnPP2C* family members. The hormones involved in the phytohormone response elements were MeJA, ABA, and GA, etc., among which ABRE had the largest number, followed by the CGTCA-motif and the TGACG-motif. The *cis*-elements involved in stress were less diverse but more numerous, mainly including *cis*-elements that are responsive to low temperature, hypoxia and drought stress, such as ARE elements. These results suggest that *BnPP2Cs* might play important regulatory roles in response to light regulation, hormone signaling and adversity stress.

### 2.6. Expression Profiling of PP2C Gene Family in Different Tissues of Ramie

**Figure 7:**
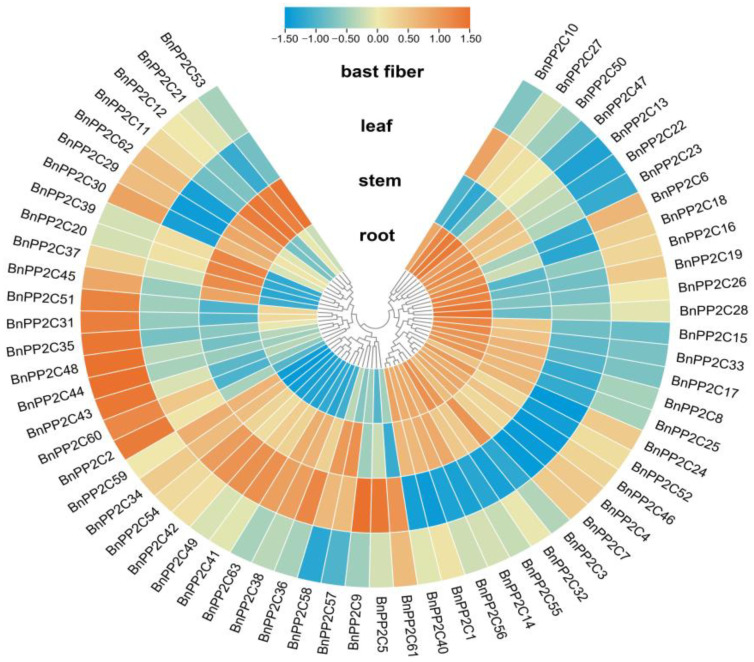
Expression profiles of 63 *BnPP2C* genes based on RNA-seq data: red and blue boxes indicate high and low expression levels of *BnPP2Cs*, respectively.

In order to gain insight into the tissue-specific expression pattern of ramie, we used the transcriptome data to create a heat map of the relative expression of *BnPP2C* in roots, stems, leaves and bast fibers (Figure 7). The results showed that *BnPP2Cs* were differentially expressed in different tissues, among which *BnPP2C51*, *BnPP2C31*, *BnPP2C35*, *BnPP2C48*, *BnPP2C44*, *BnPP2C43*, *BnPP2C60* and *BnPP2C2* were highly expressed in bast fibers, while little or no expression was found in rootstocks and leaves, indicating that these genes might be accompanied in the synthesis of bast fibers. *BnPP2C53*, *BnPP2C21*, *BnPP2C12*, *BnPP2C11*, *BnPP2C62*, *BnPP2C29*, *BnPP2C30*, *BnPP2C39*, *BnPP2C20*, *BnPP2C37* and *BnPP2C45* were highly expressed in the stems, indicating that these genes may have a regulatory role in the growth and development of ramie stems. The results implied that *BnPP2Cs* might play multiple regulatory roles in growth and development.

### 2.7. Analysis of Expression Patterns of BnPP2C Genes under Drought, Salt and ABA Treatments

**Figure 8:**
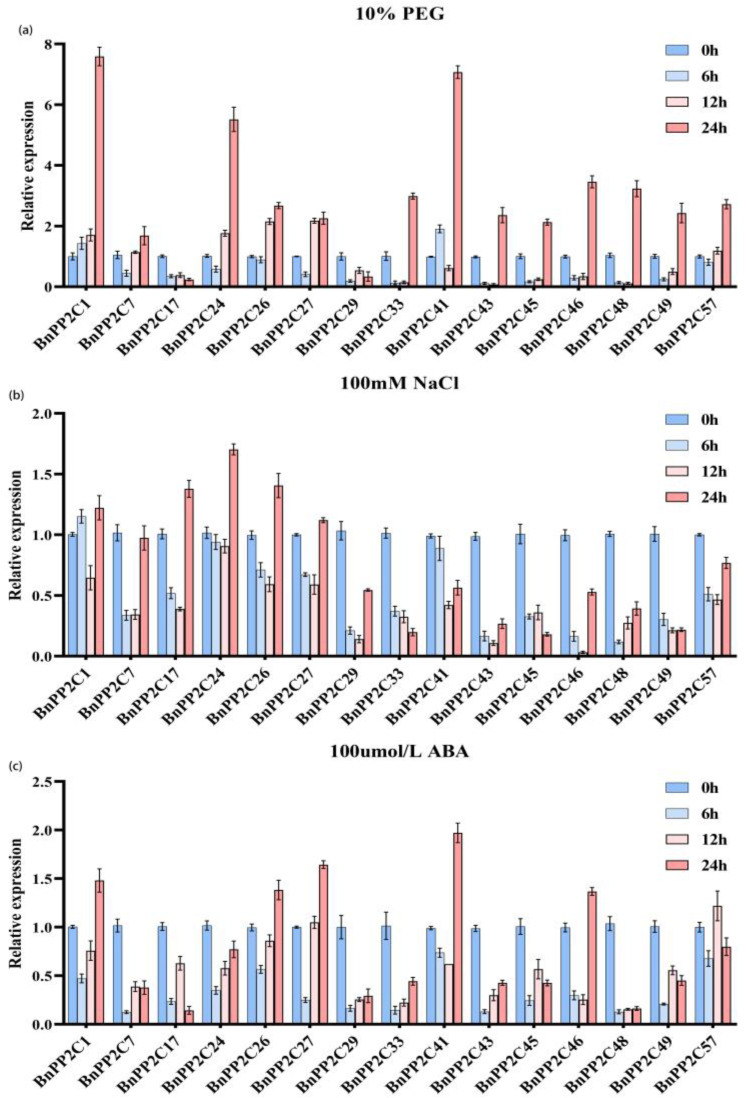
Quantitative real-time PCR analysis of *BnPP2C* genes in response to 10% PEG (**a**), 100 mM NaCl (**b**) and 100 μM ABA (**c**). Note: The relative gene expression was calculated using the 2^−∆∆Ct^ method, and its value is the average of three biological replicates.

The analysis of the promoter *cis*-acting element of *BnPP2Cs* suggested that this gene family might play an important role in response to abiotic stresses and hormonal regulation. Therefore, qRT-PCR was used to analyze the expression of 15 of these *BnPP2C* genes under ABA, PEG and salt treatments. The quality testing of RNA mainly includes concentration, purity and integrity testing, the results of which play an important role in the subsequent experiments. The concentration of RNA in this test was between 950 and 1200 ng/μL, and the OD260/OD280 were all around 2.0. Agarose electrophoresis found three bands, and the 28s and 18s bands were obvious, which indicated the integrity was better. The above results confirmed that the quality of the RNA was reliable and could be used for the subsequent experiments, and the results of its test are shown in the Appendix (Supplementary Table S6 and Supplementary Figure S1). Dissolution curve analysis is a method to verify primer specificity. We carried out the dissolution curve analysis and found that the dissolution curves of each primer were single peaks and that the Tm values were all greater than 80 °C, indicating that the primers were specific; the results are shown in Supplementary Figure S2. The 15 selected genes showed high homology with 15 of the cucumber *PP2C* family members, and the expression of these cucumber *PP2C* members were identified to be significantly up-regulated under drought, salt stress and ABA treatments. The treatment was conducted with 10% PEG, 100 mM NaCl and 100 μM ABA, as pre-experiments and references suggested [7,12]. The results showed that the expression of all of the 15 selected *BnPP2C* genes were down-regulated within 6 h of ABA treatment, but the expression of the *BnPP2C1*, *24*, *26*, *27*, *29*, *33*, *43* and *48* genes gradually increased as the treatment time extended, with the highest expression level of *BnPP2C41* at 24 h, which was about twice as high as that at 0 h. ABA suppressed the expression of *BnPP2C48* genes at 24 h (Figure 8). 

Under PEG-induced drought stress, the expression of all 14 *BnPP2C1* genes were down-regulated at 6 h except for *BnPP2C1*, but the expression of *BnPP2C7*, *24*, *26*, *45*, *46*, *49* and *57* gradually increased with an increase in the stress time. Among them, the expression of *BnPP2C1* gradually increased from the onset of stress, and the highest expression was at 24 h, which was about 7.5 times of that at 0 h. After 6 h of salt stress, only the expression of *BnPP2C1* was up-regulated. After 12 h of salt stress, the expression of the 15 selected *BnPP2C* genes were all down-regulated compared to 0 h, with *BnPP2C46* being the most significantly down-regulated. After 24 h of stress, only the expression of *BnPP2C1*, *17*, *24*, *26* and *27* genes were up-regulated compared with CK, with *BnPP2C24* being the most significantly up-regulated, which was 1.7 times more than that at 0 h.

In summary, some genes showed similar expression patterns under PEG and ABA treatments, such as *BnPP2C24* and *BnPP2C27*, with the lowest expression at 6 h, and gradually increasing thereafter. However, under salt stress treatment, *BnPP2C24* and *BnPP2C27* had the lowest expression at 12 h, indicating that *BnPP2C* genes have different expression patterns under different stress treatments, and the above genes might be involved in the response process of ramie to drought stress, salt stress and ABA treatment.

## 3. Discussion

### 3.1. Global Profile of the PP2C Gene Family of Ramie

The *PP2C* family is one of the most numerous and widely distributed gene families in plants, which plays important roles in coping with abiotic stresses such as drought and salinity, as well as in plant growth and development [27,28,29]. *PP2C* is known to be mainly involved in stress signaling, but most of the functions of *PP2C* remain undefined. Although the *PP2C* gene family has been extensively studied in other species, little research has been undertaken in ramie [30]. In this study, the genome-wide identification and screening of *PP2C* genes were carried out, followed by analyses of phylogenetics, gene structure, gene localization, covariate relationships, *cis*-acting elements, tissue expression, and the expression pattern under abiotic stresses. It laid an important foundation for understanding the potential molecular mechanism of ramie *PP2C* genes in the signal transduction of adversity and stress.

The number of *PP2C* gene family members may be related to the genome size of the species or may have changed during the course of evolution [31]. In the present study, 63 *BnPP2C* genes were identified and categorized into 12 subfamilies. Interestingly, none of the BnPP2C genes have been categorized into subgroup K, and no details have been reported on the function of PP2C subfamily K in other species [5]. The physicochemical properties of the members of the PP2C family of ramie differ among subfamilies, and most of them are acidic proteins. Protein localization predictions showed that the *BnPP2C* genes were mainly localized in chloroplasts and cytoplasm, which might be related to the anabolism of photosynthetic enzymes and to cell growth and development. Members of the same subgroup of the *BnPP2C* gene have similar exon–intron structures, and the proteins they encode have similar motif components [12,23]. In cucumber, the vast majority of *CsPP2C* genes were present in Motif 1 and 2, whereas in ramie the vast majority of *BnPP2C* genes were present in Motif 4, 7 and 9 structures. This pattern of motif arrangement may be closely related to the catalytic core structural domains of PP2C proteins, and further confirms that the PP2C gene family is highly conserved in evolution [32]. We also found the presence of PKc-like superfamily structural domains in BnPP2C28, 38 and 59, which may play a role in the signaling cascade response [33].

The phenomenon of gene duplication was one of the most important reasons for the diversification of gene functions, and segmental and tandem duplication were the main drivers of the evolution and expansion of family genes [34,35]. This might also be one of the reasons for the difference in the number of members of the *PP2C* gene family in ramie compared to other plants. The presence of three pairs of tandem-duplicated genes and seven pairs of segmental-duplicated genes among the ramie *PP2C* family suggests that segmental duplication plays a major role in the amplification of the *BnPP2C* gene family, which was consistent with the major amplification of the rice *PP2C* gene in *Arabidopsis* [5]. The results of Ka/Ks analysis showed that, except for two pairs of genes with significant sequence differentiation and a long evolutionary distance, the Ka/Ks values of the rest of the *BnPP2C* gene pairs were all less than 1, suggesting that the functional differentiation of ramie *PP2C* family genes was limited; similar results were found in woodland strawberry (*Fragaria vesca)*, pineapple strawberry (*Fragaria ananassa)*, cotton (*Gossypium hirsutum*) and tomato (*Solanum lycopersicum*) [12,36,37].

### 3.2. The Roles of the BnPP2C Gene Family in Response to Abiotic Stresses

Currently, drought and salt stress have become the key factors limiting the productivity of ramie, and the excavation of key drought and salt tolerance genes is important for the advancement of ramie breeding programs [38,39]. It was shown that some *PP2C* genes in both cucumber and wheat showed a tendency to be up-regulated in expression after treatment with drought, salt stress, or ABA, which was consistent with the results of this study on ramie [7]. In walnut, *JrPP2C17* showed a strong response to drought, high salt and ABA [8]. GlPP2C1 silencing increased the content of *Ganoderma lingzhi* polysaccharides (GL-PS) through Slt2, thereby enhancing cell wall resistance [40]. Some genes may have opposite roles in different plants. Compared with the wild type, the enhanced drought tolerance of the maize *Zmpp2c26* mutant is mainly regulated by the dephosphorylation of *ZmMAPK3* and *ZmMAPK7* by *ZmPP2C26*. However, overexpression of *ZmPP2C26L* and *ZmPP2C26S* significantly reduced drought tolerance in *Arabidopsis* and rice [41]. The qRT-PCR results in this study showed that some *BnPP2C* genes showed similar expression patterns under PEG and NaCl treatments, such as *BnPP2C1*, *BnPP2C24*, *BnPP2C26* and *BnPP2C27* genes, presenting a U-type curve. Interestingly, the expression of *BnPP2C24* decreased and did not change significantly under ABA treatment for 24 h, suggesting that the *BnPP2C24* gene might be a negative regulator of the ABA-mediated signaling pathway. However, *BnPP2C1*, *BnPP2C 26* and *BnPP2C27* genes may positively regulate the ABA signaling pathway in response to adversity stress, thereby enhancing ramie resilience; similar results were observed in cucumber [7].

*Cis*-acting elements are important regulators of plant hormone responses and resistance to various adversities [42]. *Cis*-acting elements were detected to be responsive to various phytohormones, such as MeJA, GA, ABA, IAA and SA, in the promoter regions of *PfPP2C* genes in *Paulownia fortunei* [31]. The multi-roles of PP2C genes were also reported in *A. thaliana* [43], *Saccharum spontaneum* [3] and other species. The promoter regions of *BnPP2Cs* were enriched with light-responsive elements and hormone-responsive elements, such as G-box, Box4, P-box, ABRE, etc., which suggested that *BnPP2Cs* might play important roles in responding to photoperiodic and hormone-induced processes [44]. Among them, there were also multiple stress-responding elements in the promoter regions of *BnPP2C1* and *BnPP2C26*, and these results supported the fact that both *BnPP2C1* and *BnPP2C26* may enhance ramie resistance by participating in the ABA signaling pathway.

### 3.3. Candidate Genes for Improving Drought and Salt Tolerance

Designing crops that are more productive, coupled with being more environmentally resilient, is a major goal of breeding scientists. Understanding the synergies between crop yield and stress response is critical to developing new varieties [45,46]. Abiotic stresses can lead to ramie root failure, slow growth, leaf loss and other undesirable phenomena, and finally impact fiber quality and yield [47,48]. As the global climate and environment degenerate, multi-stress conditions, especially drought coupled with salt stress, have become more and more frequent in agricultural production, especially affecting food crop [49], fiber crop [50] and horticulture crop [51].

In this study, we found a large number of stress-responsive elements in the promoter regions of *BnPP2C1* and *BnPP2C26*, which were positively regulated in the ABA signaling pathway and abiotic stress response mechanisms. In summary, *BnPP2C1* and *BnPP2C26* can be used as candidate genes to improve salt and drought tolerance in ramie, but further studies are needed to reveal their specific functional mechanisms in ramie growth and resistance to adversity stress.

## 4. Materials and Methods

### 4.1. Identification of BnPP2C Family Members and Analysis of Physicochemical Properties

The *BnPP2C* family members were identified from the whole-genome data of ramie in our laboratory. The hidden Markov model (HMM) mapping of the *PP2C* structural domain (PF00481) was first obtained through the Pfam database (http://pfam.xfam.org/, accessed on 5 April 2023) and we downloaded the *PP2C* protein sequence of *Arabidopsis* from the TAIR website (The *Arabidopsis* Information Resource, https://www.Arabidopsis.org/, accessed on 5 April 2023). The target genes were searched using HMMER3.0 software with a threshold E-value ≤ 1 × 10^−5^ [52,53]. The protein sequences of the candidate *BnPP2C* genes were submitted to CD-search (https://www.ncbi.nlm.nih.gov/cdd/, accessed on 7 April 2023) and SMART (http://smart.embl-heidelberg.de/, accessed on 7 April 2023) for protein structural domain analysis, removing redundancies and retaining protein sequences containing *PP2C* structural domains [54].

The physicochemical properties of *BnPP2C* proteins, including amino acid number, molecular weight and theoretical pI, were predicted by ExPASy ProtParam (https://web.expasy.org/protparam/, accessed on 10 April 2023) [55]. The subcellular localization of *BnPP2C* proteins was predicted by the Wolf PSORT website (https://wolfpsort.hgc.jp/, accessed on 10 April 2023) [56].

### 4.2. Phylogenetic Tree Construction of the BnPP2C Gene Family

The 63 *BnPP2C* and 80 *AtPP2C* protein sequences were imported into MEGA11 software together; the ClustalW method was used for multiple sequence alignment, and the neighbor joining method was used to construct a phylogenetic tree (the bootstrap value was set to 1000 and other parameters were set as default) [57]. The ramie *PP2C* proteins were subdivided into different subfamilies, according to the classification of *AtPP2Cs*. The phylogenetic tree was embellished and visualized using the online software iTOL (https://itol.embl.de/, accessed on 12 April 2023) [58].

### 4.3. Analysis of BnPP2C Gene Structure, Conserved Motifs and Conserved Structural Domains

The conserved patterns (CMs) of *BnPP2C* proteins were predicted on the MEME Suite 5.4.1 online tool (https://meme-suite.org/meme/tools/meme/, accessed on 14 April 2023) with the conserved pattern parameter set to 10 and other parameters set as default. The Gene Structure View function in TBtools was used to generate an overall map of the conserved patterns, conserved structural domains and gene structures of *BnPP2Cs* [59,60].

### 4.4. Chromosomal Localization and Covariance Analysis of BnPP2C Gene

The chromosome length information and PP2C gene position information in the chromosome were extracted from the ramie genome annotation file using TBtools software, and the family members are named *BnPP2C1* to *BnPP2C63* based on their location on the chromosome [61].

Gene duplication between ramie *PP2C* family genes within and between species (ramie and *Arabidopsis*) was analyzed using MCScanX and visualized using Advanced Circos. Using the KaKs_Calculator 2.0, we computed the synonymous (Ks) and non-synonymous (Ka) substitutions of the *BnPP2C* gene pairs [62,63].

### 4.5. Analysis of Cis-Acting Elements of the BnPP2C Gene Family

The first 2000 bp of upstream sequences of the start codon of the 63 identified ramie *PP2C* genes were extracted using TBtools for *Cis*-acting prediction through the PlantCARE database (http://bioinformatics.psb.ugent.be/webtools/plantcare/html/, accessed on 17 April 2023) and their potential related functions, and the number of each acting element was counted by Excel 2019 [64].

### 4.6. Tissue-Specific Expression Analysis of the BnPP2C Gene Family

The specific expression of *BnPP2C* gene in different tissues (root, stem, leaf and bast) was investigated using the ramie transcriptome database “Zhongzhu No. 1” in our laboratory, and the expression heat map of *BnPP2C* gene in four different tissues was drawn using TBtools [61].

### 4.7. Plant Materials and Growth Conditions

Ramie seeds were rinsed well, soaked in pure water for 10 min, and evenly beaten on the surface of nutrient soil with a pipette gun, then covered with about 1cm of soil, watered and placed in a greenhouse (humidity (60 ± 5%), temperature (30 ± 2 °C)). When the ramie seedlings reached four true leaves, we selected seedlings of roughly uniform growth and transplanted them into the hydroponics apparatus for half a month, during which the nutrient solution was added. We set up three treatment groups (T1: 100 mM NaCl; T2: 100 μM ABA; T3: 10% PEG) and one control group, with six biological replicates for each treatment, and the stress conditions were selected with reference to the expression pattern analysis of the *PP2C* family members in cucumber. Ramie leaves after 0, 6, 12 and 24 h of different treatments were taken, wrapped in tinfoil, placed in liquid nitrogen and stored in a −80 °C refrigerator.

### 4.8. RNA Sample Extraction and qPCR Analysis

We performed qPCR analysis using samples from the above three treatments and four time periods. Firstly, gene-specific primers were designed through the Primer-BLAST online website (https://www.ncbi.nlm.nih.gov/tools/primer-blast/index.cgi?LINK_LOC=BlastHome, accessed on 29 April 2023). The SteadyPure Plant RNA Extraction Kit (Accurate Biotechnology (Changsha, China) Co., Ltd.) and Evo M-MLV RT Premix for qPCR Kit (Accurate Biotechnology (Changsha, China) Co., Ltd.) were used for total RNA extraction and reverse transcription. RNA concentration and purity were measured using a NanoDrop 2000 spectrophotometer (Thermo Scientific, Wilmington, DE, USA), and RNA integrity was analyzed using 1% agarose gel electrophoresis. The 18s gene was used as an internal control (accession number: EU747115) and qRT-PCR was performed using a 2× SYBR Mixture (Biomiga(Beijing, China) Co., Ltd.) fluorescence quantification kit. The reaction system was as follows: 2× SYBR Green Pro Taq HS Premix: 5 μL, cDNA after 2000-fold dilution: 2 μL, forward and reverse primers: 0.2 μL, and ddH_2_O: 2.6 μL. The reaction procedure was as follows: pre-denaturation at 95 °C for 3 min, denaturation at 95 °C for 30 s, annealing at 56 °C for 20 s, extension at 72 °C for 15 s, 40 cycles. Three technical replicates were set for each reaction. The relative expression of the *BnPP2Cs* was calculated using the 2-ΔΔCt method and plotted using GraphPad Prism v8.0 software [65,66]. The primer sequences are shown in Supplementary Table S5.

### 4.9. Statistics and Analysis of Data

Excel 2019 was used for statistics and analysis of data, and bar charts were created using Prism software.

## 5. Conclusions

A comprehensive analysis of the *PP2C* family genes in ramie was carried out in this study. We identified 63 *BnPP2C* genes using bioinformatics methods and classified them into 12 subfamilies based on the taxonomy of *Arabidopsis thaliana*; their conserved sequence features, gene structure and motif composition consistently supported the classifications. The physicochemical properties varied among subfamilies, with the majority being acidic proteins. The subcellular localization predictions revealed that the ramie *PP2C* genes were mainly localized in the chloroplast and cytoplasm. Synteny analysis revealed that fragment replication played a major role in the amplification of the *BnPP2C* gene family. The analysis of promoter *cis*-acting elements showed that members of *BnPP2C* respond positively to plant growth, hormone signaling and stress stimuli. Based on transcriptomic data, *BnPP2Cs* were found to have significant tissue expression specificity. By analyzing the expression pattern of *BnPP2Cs*, we identified some key genes associated with drought and salt stress. In conclusion, these results provide a useful reference for further investigation of the *BnPP2C* gene functions.

## Data Availability

The datasets used and/or analyzed during the current study are available from the corresponding author on reasonable request.

## Supplementary Materials

The following supporting information can be downloaded at: https://www.mdpi.com/article/10.3390/ijms242015282/s1.
