# Supplementary material for: Genome-Wide Identification and Expression Analysis of BnPP2C Gene Family in Response to Multiple Stresses in Ramie (Boehmeria nivea L.)
Source: Int J Mol Sci. 2023 Oct 18;24(20):15282. doi: 10.3390/ijms242015282 (PMC10607689; doi:10.3390/ijms242015282)
Supplement: Supplementary file 1 [file ijms-24-15282-s001.zip › Supplementary Figure S1.pdf]

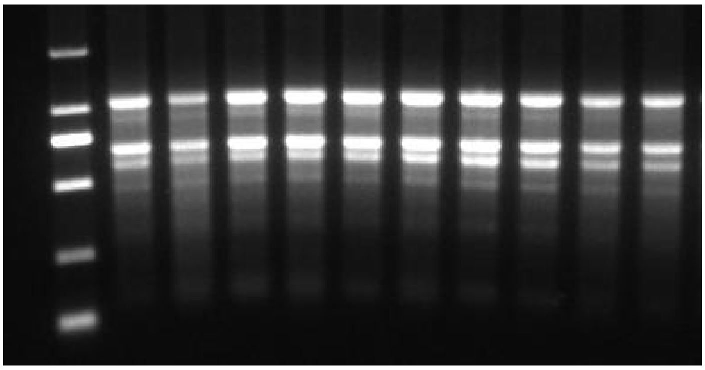

Figure S1. RNA gel electropherogram. From left to right, gel electrophoresis of PEG/ NaCl / ABA -0h, PEG-6h, PEG-12h, PEG-24h, NaCl-6h, NaCl-12h, NaCl-24h, ABA-6h, ABA-12h, ABA-24h RNA samples. The bands are 28s, 18s, and 5s in order from top to bottom.
