# Supplementary figures and images for: Genome-Wide Identification and Expression Analysis of BnPP2C Gene Family in Response to Multiple Stresses in Ramie (Boehmeria nivea L.)
Source: Int J Mol Sci. 2023 Oct 18;24(20):15282. doi: 10.3390/ijms242015282 (PMC10607689; doi:10.3390/ijms242015282)

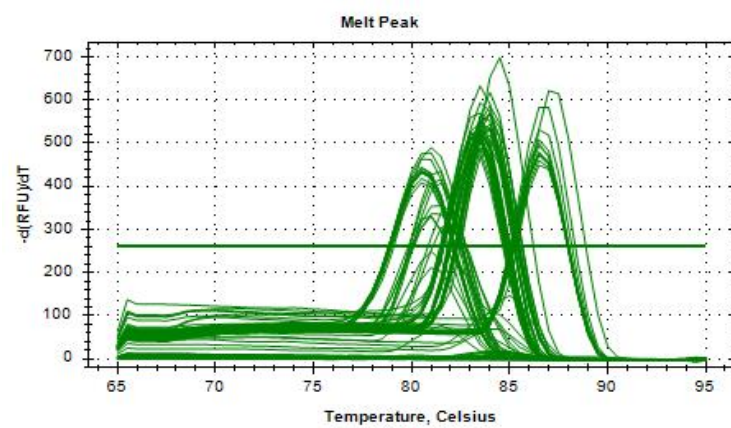

Figure S2. Melting Curve Analysis.

Supplement: Supplementary file 1 [file ijms-24-15282-s001.zip › Supplementary Figure S2.pdf]
